# Molecular insight into human platelet antigens: structural and evolutionary conservation analyses offer new perspective to immunogenic disorders

**DOI:** 10.1111/j.1537-2995.2010.02862.x

**Published:** 2011-03

**Authors:** Meytal Landau, Nurit Rosenberg

**Affiliations:** Amalia Biron Research Institute of Thrombosis and Hemostasis, Chaim Sheba Medical Center, Tel-Hashomer; and the Sackler Faculty of Medicine, Tel-Aviv UniversityTel-Aviv, Israel

## Abstract

**BACKGROUND:**

Human platelet antigens (HPAs) are polymorphisms in platelet membrane glycoproteins (GPs) that can stimulate production of alloantibodies once exposed to foreign platelets (PLTs) with different HPAs. These antibodies can cause neonatal alloimmune thrombocytopenia, posttransfusion purpura, and PLT transfusion refractoriness. Most HPAs are localized on the main PLT receptors: 1) integrin αIIbβ3, known as the fibrinogen receptor; 2) the GPIb-IX-V complex that functions as the receptor for von Willebrand factor; and 3) integrin α2β1, which functions as the collagen receptor.

**STUDY DESIGN AND METHODS:**

We analyzed the structural location and the evolutionary conservation of the residues associated with the HPAs to characterize the features that induce immunologic responses but do not cause inherited diseases.

**RESULTS:**

We found that all HPAs reside in positions located on the protein surface, apart from the ligand-binding site, and are evolutionary variable.

**CONCLUSION:**

Disease-causing mutations often reside in highly conserved and buried positions. In contrast, the HPAs affect residues on the protein surface that were not conserved throughout evolution; this explains their naive effect on the protein function. Nonetheless, the HPAs involve substitutions of solvent-exposed positions that lead to altered interfaces on the surface of the protein and might present epitopes foreign to the immune system.

Polymorphisms in platelet (PLT) membrane glycoproteins (GPs) are responsible for alloantibody production upon exposure to PLTs with different human PLT antigens (HPA). These antibodies can cause neonatal alloimmune thrombocytopenia (NAIT), posttransfusion purpura, or PLT transfusion refractoriness after exposure to unmatched PLTs. The molecular basis of the 24 serologically defined antigens had been resolved^1^ and found to be a single-nucleotide polymorphism (SNP) resulting in an amino acid substitution in 23 of 24 antigens: 12 HPAs are grouped in six biallelic systems (HPA-1 to -5 and -15) in which alloantibodies against both the common (designated a) and the rare (designated b) alleles were observed. For the rest, only alloantibodies against the rare allele have been detected. The only non-SNP is HPA-14bw, an in-frame triplet deletion coding for one amino acid (β3-Lys611).^2^ Overall, there are 17 different HPAs. A nomenclature was devised by the International Society of Blood Transfusion and the International Society of Thrombosis and Haemostasis (see Table 1).

**TABLE 1:** **Data of the HPAs**

HPA (frequency*)	Gene	SNP	Amino acid	Domain	Residue's SASA	Secondary structure	Evolutionary conservation
1(0.15)	β3	176 T>C	Leu33Pro Leu33Val	PSI	98% (3FCS)	Loop	1 = variable
		175 C>G			100% (3FCU)		
2(0.07)	GPIbα	482 C>T	Thr145Met	LRR	67% (1SQ0)	Loop	3 = variable
3(0.39)	αIIb	2621 T>G	Ile843Ser	Calf2	Disordered region (3FCS)	Loop	2 = variable
4(0.01†)	β3	506 G>A	Arg143Gln	β A	62% (1JV2)	α-Helix	2 = variable
					43% (1TXV)		
					76% (3FCS)		
					43% (3FCU)		
5(0.11)	α2	1600 G>A	Lys505Glu	β-Propeller	73% in model	Loop	1 = variable
6(<0.01)	β3	1544 G>A	Arg489Gln	EGF-1	76% (3FCS)		1 = variable
7(<0.01)	β3	1297 C>G	Pro407Ala	Hybrid	27% (1JV2)	Loop	6 = intermediate
					17% (1TXV)		
					8% (3FCS)		
8(<0.01)	β3	1984 C>T	Arg636Cys	βTD	59% (1JV2)	Loop	1 = variable
					66% (3FCS)		
9(<0.01)	αIIb	2602 G>A	Val837Met	Calf2	40% (3FCS)	Loop	6 = intermediate
10(<0.01)	β3	263 G>A	Arg62Gln	Hybrid	32% (1JV2)	β-Sheet	1 = variable
					63% (1TXV)		
					71% (3FCS)		
					69% (3FCU)		
11(<0.01)	β3	1976 G>A	Arg633His	βTD	17% (1JV2)	Loop	1 = variable
					53% (3FCS)		
12(<0.01)	GPIb β	119 G>A	Gly15Glu		No structure		2 = variable
13(<0.01)	α2	2483 C>T	Thr799Met	Calf1	32% in the model	β-Sheet	6 = intermediate
14(<0.01)	β3	1909-11 del	Lys611del	βTD	15% (1JV2)	α-Helix	4 = variable
					41% (3FCS)		
15(0.49)	CD109	2108 C>A	Tyr703Ser		No structure		4 = variable
16(<0.01)	β3	497 C>T	Thr140Ile	βA	51% (1JV2)	α-Helix	1 = variable
					31% (1TXV)		
					55% (3FCS)		
					60% (3FCU)		
17(<0.01)	α2	3389 C>T	Thr1087Met	Calf2	53 % in the model	β-Sheet	5 = intermediate

* The frequency of the rare allele in Europeans is given in parentheses.

† The frequency of the rare allele in an Asian person.

Most HPAs are localized to the main PLT receptors, namely, integrin αIIbβ3 that is also known as the fibrinogen receptor, the complex GPIb-IX-V that functions as the receptor for von Willebrand factor (VWF) and integrin α2β1—the collagen receptor. Exceptional is HPA-15, which is carried by the glycosylphosphatidylinositol-linked protein CD109^3^ that was found to be a part of the transforming growth factor (TGF)-β receptor system and functions as a negative regulator of its signaling.^4^

Integrins are adhesion receptors that mediate vital bidirectional signals within the cell.^5^ They form heterodimers of an α and a β subunit, both Type I membrane proteins with large extracellular segments.^6^,^7^ There are 24 known heterodimers in mammals, composed of 18 α and eight β subunits.^5^ The NH_2_-terminal ectodomain of the α and β subunits assembles into an ovoid “head” and two “legs” composed of several domains. The remaining segments form two tails that span the plasma membrane.^6^,^7^

Integrin β3 subunit is known as PLT membrane GPIIIa that forms heterodimers with αIIb and αv integrins subunits; αIIbβ3 is the most abundant PLT receptor with approximately 80,000 copies per PLT. The structure of the inactive extracellular region of αvβ3 was the first one reported (PDB ID 1JV2^7^). The structure of the entire ectodomain of αIIbβ3 was determined in the inactive conformation (PDB ID 3FCS^8^). The active conformation of αIIbβ3 was determined only for part of the ectodomain (e.g., PDB ID 1TXV^6^,^9^ and 3FCU^8^). For the structural analyses, we used the crystal structures relevant to each of the HPAs.

Eleven HPAs are located on the most abundant receptor on PLTs surface: integrin αIIbβ3. Nine of the HPAs are mapped to the β3 subunit that was found to be the most polymorphic GP on the PLT membrane.^10^ Some of the HPAs are frequent in the Caucasian population (HPA-3 and HPA-15) but most of them are rare and some are private polymorphisms, restricted to one family (http://www.ebi.ac.uk/ipd/hpa/freqs_1.html). The antigen most commonly implicated in alloantibodies production is HPA-1a, a Leu33Pro substitution in integrin β3. Approximately 80% of NAIT cases are caused by anti-HPA-1a. The second frequent antigen causing NAIT is HPA-5b (10%-15% of NAIT cases), while all the rest cause NAIT very rarely.^11^ Only 10% of HPA-1b–homozygous women with an HPA-1a–positive fetus develop anti-HPA-1a alloantibodies during pregnancy.^12^ Maternal responsiveness to HPA-1a shows strong association with DRB*0101 allele encoding human histocompatibility leukocyte antigen DR52a. It was shown that short peptide containing HPA-1a (Leu33) bind to recombinant DR52a molecules, whereas the HPA-1b (Pro33) version does not.

Interestingly, although most HPAs are nonsynonymous SNPs and HPA-14w is even a single-amino-acid deletion, they probably inflict a minor effect on PLT receptors function since they do not cause inherited diseases, and even their influence on thrombus formation is controversial.^13^ The major complication is their involvement in immunogenic response.

In this article we analyzed the structural features and evolutionary conservation of the HPAs to define the characteristics that lead to immunologic problems but not to inherited disease. We demonstrate that all HPAs studied here involve residues located on the surface of the protein far from the ligand-binding site and are not evolutionary conserved. These criteria imply that the substitutions in these positions present a minor effect on the structure and function of the protein and therefore, do not represent disease-causing mutations. Moreover, their location on the surface suggests that they could play a role in presenting epitopes.

## MATERIALS AND METHODS

### Evolutionary conservation analyses

The evolutionary conservation analyses were calculated using the Bayesian method^14^ implemented in the ConSurf Web server (http://consurf.tau.ac.il)^15^ or the ConSeq Web server (http://conseq.tau.ac.il/).^16^ The conservation scores range from 1 to 9, indicating variable-to-conserved positions.

#### α-Integrins

A multiple sequence alignment (MSA) of the β-propeller domain in various α-integrins was constructed as described previously.^17^ This alignment was used to calculate the conservation scores for the HPAs in the β-propeller domain of α2. An MSA of the thigh and calf domains was taken from the α-integrin family in the Pfam database (http://pfam.sanger.ac.uk/). This alignment corresponds to Residues 481 to 921 in αIIb and was used to calculate the conservation scores for the HPAs in the calf domain of αIIb. The last two strands of the calf2 domain constitute a very variable region among α-integrins and therefore were not included in the Pfam alignment. Since one of the HPAs in α2 is located on the C-terminus of the calf2 domain, we generated a smaller MSA via ConSeq, using α2 human as the query sequence to collect 50 homologs from the Swiss-Prot database^18^ that were aligned using MUSCLE.^19^ We note that the conservation scores for HPA-13 located in calf1 were identical when using in the Pfam and ConSurf generated alignment.

#### β-Integrin

Human β3 (Swiss-Prot entry ITB3_HUMAN) was used as query to collect homologous sequences from the Swiss-Prot database^18^ using PSI-BLAST.^20^ The resulted 27 sequences were aligned using CLUSTALW^21^ with default parameters. This intermediate alignment was used to generate a hidden Markov model,^22^ which was subsequently utilized to collect remote homologous sequences from the UniProt database^23^ that were aligned using MUSCLE.^19^ From the 105 hits found, redundant (above 95% sequence identity) and fragmented sequences, as well as sequence variants and mutants, were discarded along with sequences that included irregular characters or ones that were sequenced by the whole genome shotgun project therefore considered as preliminary data. The resulted alignment contained 66 β-integrins and was used to calculate the evolutionary conservation scores in β-integrins. To support the analysis, we also used an alignment generated by ConSurf using 300 homologous sequences collected from the UniProt database^23^ and aligned using MUSCLE.^19^ The conservation scores were very similar.

#### Leucine-rich repeat family of proteins

Human GPIbα (PDB ID 1SQ0, Chain B)^24^ was used as query to calculate the evolutionary conservation scores via the ConSurf Web server.^15^ The calculations were based on an MSA constructed from 200 homologous sequences from the Swiss-Prot database^18^ collected using PSI-BLAST and aligned with MUSCLE.^19^ Since the structure of human GPIbβ (Swiss-Prot entry: GP1BB_HUMAN) is not available, we used the ConSeq Web server^16^ to calculate the evolutionary conservation scores using 90 sequences collected from the Swiss-Prot database with PSI-BLAST^20^ and aligned by MUSCLE.^19^

#### CD109

Since the structure of human CD109 is not available (Swiss-Prot entry: CD109_HUMAN), the ConSeq Web server^16^ was used to calculate the conservation scores of human CD109 based on 66 sequences collected from the Swiss-Prot database^18^ using PSI-BLAST^20^ and aligned by MUSCLE.^19^

### Structural analyses

The analyses were based on the crystal structures of αVβ3 (PDB ID 1JV2),^7^αIIbβ3 (PDB ID 1TXV,^6^,^9^ 3FCS,^8^ and 3FCU),^8^ and GPIbα (PDB ID 1SQ0).^24^ The structure of α2 was modeled based on the crystal structure of αIIbβ3 (PDB ID 3FCS^8^) using NESTs^25^ with default parameters. The alignment between αIIb and α2 was derived from an MSA of 50 homologs sequences of α-integrins generated by ConSurf as described above. The β-propeller domain, lacking the I domain, was modeled separately using an alignment with αIIb derived from an MSA of the β-propeller domain of α-integrins constructed as described previously.^17^

The solvent-accessible surface area (SASA) was calculated using the SURFV program with a probe sphere of radius 1.4 Å and default parameters.^26^ The percentage of the surface exposure of each residue in the monomer was calculated from the total solvent-accessible area on a Gly-X-Gly tripeptide (where X represents each of the 20 amino acids). We consider a residue to be buried if less than 5% of its surface is accessible to the solvent.^27^ More importantly, we examined the location of the residue in relation to the surface of the protein.

### Statistical analysis

Data are expressed as mean ± standard deviation. Kruskal-Wallis test was used to analyze the difference between groups using computer software (GraphPad PRISM5, GraphPad Software, Inc., San Diego, CA). A p value of less than 0.05 was considered as a significant difference. In case that the SASA was calculated in various structures, the mean value was used for the statistical analysis.

## RESULTS

### HPA polymorphisms in integrin β3

Integrin β3 ectodomain is composed of eight domains—the plexins, semaphorins, and integrins (PSI); βA; hybrid; four epidermal growth factor (EGF) domains; and a β-tail domain (βTD). There are nine HPAs located in integrin β3; seven of them (HPA-4, -7, -8, -10, -11, -14, and -16) are visible in the inactive structures of both αvβ3 (PDB ID 1JV2) and αIIbβ3 (PDB ID 3FCS). Structural analyses of the crystal structures revealed that all HPAs are exposed to the solvent (Table 1) and, moreover, are located on the surface of the protein and thus do not play a role in the stabilization of the protein fold.

Integrin activation is a multistep process that involves transitions between multiple conformations.^6^ Correspondingly, the heterodimer is highly flexible. The αvβ3 and αIIbβ3 crystal structures might have captures slightly different conformations of the β3 that led to differences in the solvent accessibility of the HPA-related residues (Table 1).

Some HPAs are located close to the interface with adjacent domains on the β subunit. For example, Arg633, the polymorphic residue of HPA-11, and Arg636, the polymorphic residue of HPA-8, both located in the βTD, are close to the hybrid domain. In the inactive complex αVβ3, Arg633 forms a salt bridge with Asp393 from the hybrid domain and is surrounded by Leu389, Gly388, and Cys374. In the structure of αIIbβ3, also in the inactive conformation, there are some conformational variations with respect to the inactive β3 in the αVβ3 complex. In αIIbβ3, Arg633 is facing away from the hybrid domain, yet might be still bound with Glu378 on the hybrid domain. Arg636 is in close proximity to Met387 and Gly388 from the hybrid domain in the αIIbβ3 complex, while in the αVβ3 structure, Arg636 is close to Asn376. Arg489 of HPA-6, located on the EGF-1 domain, is close to the Calf1 domain of the αIIb subunit (in the inactive conformation) but is not in direct contact.

**Fig. 1:**
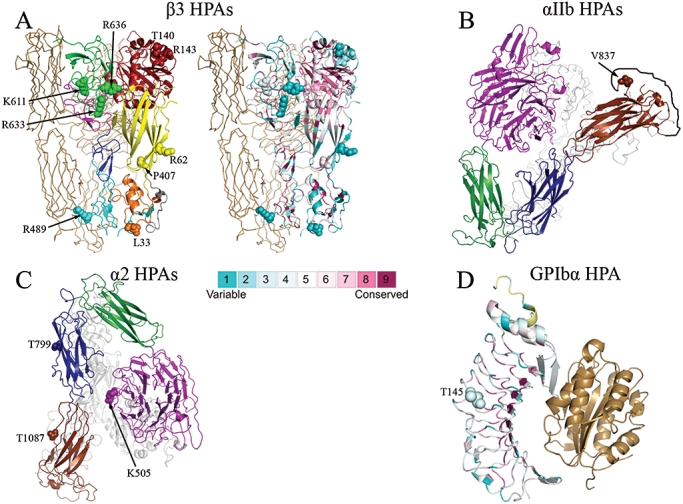
**Structural location of the HPA-related polymorphic residues. A cartoon representation of proteins involved in HPAs. The orientation was picked to best display all HPA-related residues. Scale bar for the evolutionary conservation scores is displayed in the middle. (A) HPAs at the integrin β3 subunit. The ectodomain of the αIIbβ3 complex is presented at the inactive conformation (PDB ID 3FCS****^8^****). The β3 subunit is in a cartoon representation and αIIb is presented as a sand-colored ribbon. In the left panel, β3 is colored according to the different domains—the PSI, βA, hybrid, EGF-1 to -2, EGF-3, EGF-4, and the βTD in orange, dark red, yellow, cyan, blue, magenta, and green, respectively. In the right panel, β3 is colored according to evolutionary conservation scores calculated based on a set of 66 homolog sequences of β-integrins. The HPAs polymorphic residues located in β3 are presented as space-filled atoms. It is clear that the HPAs are located on the surface of the protein and that they are all evolutionary variable. Also, all HPAs are facing away from the interface with the α subunit, suggesting that the substitutions do not disturb the heterodimeric interactions. (B) HPAs at the integrin αIIb subunit. The ectodomain of the αIIbβ3 complex is presented at the inactive conformation (PDB ID 3FCS). αIIb is in a cartoon representation, colored according to the different domains—β-propeller, thigh, calf1, and calf2 in purple, dark green, dark blue, and brown, respectively. β3 is presented as a gray-colored ribbon. Val837, associated with HPA-9, is presented as space-filled atoms. It is clear that HPA-9 is located on the surface, far from the interface with the β subunit. Ile843, associated with HPA-3, is located in a disordered region, illustrated with a black line, which could not be determined in the structure. (C) HPAs at the integrin α2. A model of α2, without the I-domain, was built using αIIb as a template. The β-propeller domain and the thigh and calf domains were built separately, to ensure the reliability of the alignment between α2 and αIIb. α2 is in a cartoon representation, colored according to the different domains—β-propeller, thigh, calf1, and calf2 in purple, dark green, dark blue, and brown, respectively. The HPAs located in α2 are presented as space-filled atoms. α2 is naturally interacting with the β1 subunit. Taking into account that the α2β1 interactions are similar to the heterodimeric interactions within the αIIbβ3 complex, the β subunit is illustrated accordingly. All HPA polymorphic residues in α2 are located on the opposite side from the interface with the β subunit. (D) HPAs at GPIbα. The GPIbα, in a complex with VWF (PDB ID 1SQ0), is shown in a cartoon representation. VWF domain is sand-colored, while GPIbα is colored according to evolutionary conservation scores. Thr145, associated with HPA-2, is presented as space-filled atoms. It is clear that HPA-2 polymorphic residue is evolutionary variable, surface exposed, and located far from the interface with the ligand VWF.**

Five polymorphic residues involved in the formation of HPA-1, -4, -7, -10, and -16 are visible in the crystal structures of both the active (PDB IDs 3FCU^8^ or 1TXV^6^,^9^) and the inactive (PDB ID 3FCS)^8^ conformations of integrin αIIbβ3, enabling analysis in both states. Different solvent exposures of HPA-4 and -7 were detected due to structural changes in the backbone between the two conformations, as well as different rotamers (side-chain conformation; Table 1). Nevertheless, in both states, all HPA-related residues were found to be located on the surface of the protein, exposed to the solvent. Moreover, all of them are located far away from the ligand binding site and are not in direct contact with the α subunit in the structurally determined conformations (Fig. 1A). Interestingly, Leu33 of the most immunogenic HPA-1a is totally exposed (Table 1) and found on the tip of a loop connecting two antiparallel β-sheets in the PSI domain. This loop is flexible and particularly long in integrins.^6^,^7^ The loop, and particularly Leu33, is close to the EGF domains in the inactive conformation (Fig. 1A).

Evolutionary conservation analysis of β-integrins from different species revealed that most HPAs occupy evolutionary variable positions (Table 1). Moreover, some of the polymorphic residues (HPA-1b, -4b, -6b, -10b, and -11b) can be found in the corresponding positions in other β-integrins (Table 2 in bold), supporting the benign effect of the amino acid substitutions on PLTs normal function. The third allele in HPA-1 (Leu33Val) is a particularly naive substitution that was also found in other β-integrins, showing a conservative change, in an exposed location. Interestingly, differences in reactivity of anti HPA-1a antibodies were observed, some bind also to β3-Val33 while others showed significantly reduced reactivity.^28^

**TABLE 2:** Amino acid residues occupying the HPA-related positions in β-integrins*

HPA	Position	Common allele	Rare allele	Residue variety in β-integrins
1	**33**	Leu	Pro (Val)	A, D, E, G, K, **L**, M, N, **P**, Q, S, T, **V**, Y
4	**143**	Arg	Gln	A, E, G, K, N, **Q, R**, S, T, V
6	**489**	Arg	Gln	A, D, E, G, H, K, N, P, **Q, R,** S, T, V
7	**407**	Pro	Ala	D, E, H, I, K, L, **P**, Q, R, S, T, V
8	**636**	Arg	Cys	D, E, G, H, K, L, N, P, Q, **R**, S, T, Y
10	**62**	Arg	Gln	D, E, F, H, I, K, L, N, **Q, R**, S, T, V
11	**633**	Arg	His	A, D, E, G, **H**, K, L, N, Q, **R**, S, T, V
14	**611**	Lys	del	D, F, H, **K**, L, N, Q, R, S, Y
16	**140**	Thr	Ile	A, D, E, G, K, L, N, Q, R, S, **T**, V

* The residue variety was gathered using the ConSurf server and was based on an alignment of β subunit integrins. For each HPA-related position, the identity of the amino acid in each of the 66 homolog sequences in the alignment was examined. A list of all possible amino acid identities for the specific position is reported. The amino acid identity occupying the positions in human β3 HPAs is in bold.

The residue related to HPA-7 (Pro407) is unique among other HPAs because it shows an intermediate evolutionary conservation. Nonetheless, this position can be occupied by a variety of amino acids and is not restricted to proline in β-integrins from other species (Table 2). Pro407 is located on a loop at the hybrid domain, facing the solvent, and is not in close proximity to other domains in the structurally determined conformation of integrin β3. The substitution (Pro407Ala) probably has a minor effect on the structure.

HPA-14, the single-deletion polymorphism, Lys611del, is located within the βTD, at the C-terminus of the ectodomain. The region encompassing Lys611 forms a helix in the αVβ3 structure; thus such a deletion might disrupt the structure. However, this helix is partially unwound in the αIIbβ3 inactive conformation (PDB ID 3FCS) raising uncertainties about its stability and contribution to the overall fold.

Recently, two additional low-frequency SNPs, not yet approved as HPAs, were reported within integrin β3: Lys137Gln and Glu628Lys.^29^ Both positions are highly evolutionary variable and located on the surface of the protein (Fig. 1A), consistent with the other HPAs.

### HPA polymorphisms in integrin αIIb

Integrin αIIb (PLT membrane GPIIb) comprises the β-propeller, thigh, and two calf domains. Two HPAs (HPA-3 and -9) were found in the αIIb integrin. Both related residues (Ile843 and Val837, respectively) are located in the calf2 domain (Fig. 1B), within a highly evolutionary variable region that shows many insertions and deletions among α-integrins. This region is disordered in the crystal structures, suggesting that it is flexible. Val837 is visible in the inactive structure of αIIbβ3.^8^ It is located on a loop facing the solvent, far from the β subunit or other domains in αIIb (Fig. 1B). Residues 840 to 873 are missing in the crystal structure of αIIbβ3 (due to the disorder of that region); thus we could not examine Ile843.

Recently, a low-frequency HPA was found in αIIb corresponding to Thr619Met substitution^29^ at the calf1 domain. As observed for the other HPAs, this position is highly evolutionary variable (ConSurf score = 1; see Materials and Methods) and located on the surface of the protein (Fig. 1B).

### HPA polymorphisms in integrin α2

Integrin α2 (PLT membrane GPIa) forms hetrodimers with integrin β1 and, similarly to the integrins discussed above, is important for PLT function. Three polymorphic residues in the α2 integrin form antigens, HPA-5, -13, and -17, localized to the β-propeller, calf1, and calf2 domains, respectively. Since the only crystal structure of α2 is for the I-domain in the β-propeller (PDB ID 1AOX^30^), we modeled the rest of the structure using αIIb (PDB ID 3FCS)^8^ as a template.

The second common allele causing NAIT (HPA-5b), affecting Lys505, is located on one of the loops of the β-propeller domain, on the surface of the protein, far from the interface with the β subunit (Fig. 1C). Lys505 corresponds to Arg355 in αIIb; this position, as well as the surrounding residues on the loop, is evolutionary variable (Table 1). The rare HPA-13b is related to the substitution of Thr799 located within a β-strand in the calf1 domain, facing the solvent (Fig. 1C). This position, which corresponds to Asp636 in αIIb, shows intermediate conservation, yet is occupied by the polymorphic methionine in other α-integrins, suggesting tolerance to such substitution. HPA-17, affecting Thr1087, is located at the C-terminus of the calf2 domain. Thr1087 shows intermediate conservation among the closest 50 sequences homologous to the human α2 (Table 1), yet is located in a region that is highly variable between α-integrins. Integrin α2 forms a complex with β1. Taking into account that the interactions between the α and β subunits resemble those of αIIbβ3 and αVβ3, all three HPAs on α2 are located on the opposite side from the interface with the β subunit.

### HPA polymorphisms in the GPIb-V-IX complex

**Fig. 2:**
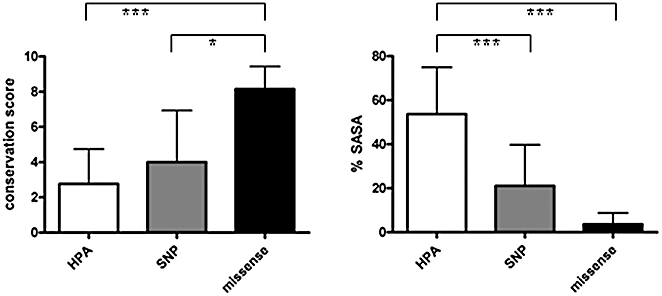
Characterization of HPAs, disease-causing missense mutations, and nonimmunogenic SNPs. Conservation score (n = 17) and residue's SASA (n = 14) of HPA-related polymorphic residues (□), missense mutations causing disease (n = 13,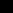), and nonimmunogenic polymorphism (n = 8, 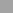) presented as mean ± SD. Significant differences marked as * p < 0.05 and ***p < 0.001.

The GPIb-V-IX complex, another PLT receptor, is composed of four Type I membrane spanning proteins that belong to the leucine-rich repeat (LRR) family of proteins: GPIbα, GPIbβ, GPIX, and GPV. HPA-2 and HPA-12 are localized to the two first GPs, respectively. Crystal structures are available only for the LRR domain of GPIbα (e.g., PDB ID 1SQ0). HPA-2, affecting Thr145, is located in this region, on the surface of the protein, facing the solvent. It is located far from the interface with its ligand, the VWF (Fig. 2D).

The GPIb-V-IX complex is known to regulate thrombin function by distinct interactions.^31^ There are two crystal structures of thrombin bound to GPIbα (PDB IDs 1P8V^32^ and 1OOK^31^). The two structures present different interpretations to how the two proteins interact.^33^ In one of these structures (PDB ID 1P8V),^32^ Thr145 is located at the interface with one of the thrombin molecules. Recently a model of GPIbβ ectodomain was constructed, based on decorin crystal structure (PDB ID 1XKU^34^), but is not yet available for analysis.^35^

### HPA polymorphisms in CD109

CD109 antigen, also known as PLT-specific Gov antigen, is a TGF-β1 binding protein for which no structure is available. The closest homolog with known structure has 26% sequence identity, which is not enough for accurate structural modeling. Tyr703 (Residue 682 in the mature protein), associated with HPA15, is evolutionary variable.

## DISCUSSION

### Polymorphisms versus disease-causing mutations

Most HPAs are localized to integrin αIIbβ3, specifically to the β3 subunit.^10^ Several missense mutations in this integrin were reported to cause severe bleeding disorder named Glanzmann thrombasthenia (GT) (http://sinaicentral.mssm.edu/intranet/research/glanzmann/menu). Single-nucleotide sequence alterations responsible for both HPAs and GT result in nonsynonymous amino acid substitutions. The interesting question is why some amino acid substitutions cause disease while others are naive polymorphisms.^36^,^37^ Moreover, some SNPs are immunogenic, while others were not reported to cause an immune response. Our working hypothesis is that mutations in residues that are evolutionary conserved and are deeply buried within the fold of the protein or located near the active site, near the ligand binding site or at the interfaces with other domains, are most likely to damage the structure and function of the protein. On the other hand, substitutions in residues that are located on the surface of the protein and are evolutionary variable will be more tolerable. Having this in mind, we examined the HPAs in terms of evolutionary conservation and structural characteristics. In comparison we analyzed mutations causing GT or Bernard-Soulier syndrome (BSS) previously reported by us (Table 3) and validated (by frequency in any population) SNPs found in NCBI SNP database that were not reported to cause an immune response (Table 4).

**TABLE 4:** **Disease-causing mutations**

Gene	Missense mutation	Amino acid	Domain	Residue's SASA	Conservation score	Disease	Reference
β3	1157 T>G	Cys13Gly	PSI	0.3% (3FCS)	9 = conserved	GT	Peretz et al.^38^
				0.64% (3FCU)			
β3	356 G>A	Arg93Glu	Hybrid	1.4% (3FCS)	9 = conserved	GT	Peretz et al.^38^
				1.4% (FCU)			
				14.5% (1JV2)			
β3	428 T>G	Leu117Trp	βA	0% (3FCS)	9 = conserved	GT	Peretz et al.^38^
				0% (FCU)			
				0% (1JV2)			
β3	652 C>T	His192Tyr	βA	4.3% (3FCS)	9 = conserved	GT	Peretz et al.^38^
				1.9% (FCU)			
				1.7% (1JV2)			
β3	1261 G>A	Val395Met	Hybrid	3.7% (3FCS)	8 = conserved	GT	Peretz et al.^38^
				1.4% (FCU)			
				4.3% (1JV2)			
β3	1723 T>C	Cys549Arg	EGF-3	13.97% (3FCS)	9 = conserved	GT	Mor-Cohen et al.^51^
				23.29% (1JV2)			
αIIb	97 A>G	Asn2Asp	β-propeller	0.55% (3FCS)	9 = conserved	GT	Mansour et al.^52^
				4.93% (3FCU)			
αIIb	416 C>T	Ala108Val	β-propeller	0% (3FCS)	8 = conserved	GT	Peretz et al.^38^
				0% (FCU)			
αIIb	607 T>G	Phe171Cys	β-propeller	24.2% (3FCS)*	8 = conserved	GT	Rosenberg et al.^17^
				23.5% (FCU)*			
				1% in the complex			
αIIb	886 G>A	Gly265Arg	β-propeller	0.1% (3FCS)	9 = conserved	GT	Peretz et al.^38^
				0% (FCU)			
αIIb	1139 G>T	Gly349Val	β-propeller	8.5% (3FCS)	8 = conserved	GT	Peretz et al.^38^
				7.8% (FCU)			
αIIb	2438 C>A	His782Asn	Calf-2	5.02% (3FCS)	6 = intermediate	GT	Losonczy et al.^53^
GPIbα	709 T>G	Trp207Gly	S-S loop	0% (1SQ0)	5 = intermediate	BSS	Rosenberg et al.^39^

* F171 is on the interface with β3. In the structure of the heterodimer, F171 is only 1% exposed to the solvent.

**TABLE 3:** **Analyses of nonsynonym polymorphisms in integrin αIIbβ3 that have not been reported as immunogenic antigens**

Gene	SNP	Amino acid	Domain	Residue's SASA	Conservation score	Database
β3	217 T>G	Leu40Arg	PSI	28.8% (3FCS)	4 = variable	rs36080296
				20.7% (3FCU)		
β3	366 C>T	Leu90Phe	Hybrid	0.56% (3FCS)	6 = intermediate	rs72547409
				0.61% (3FCU		
				1.27% (1JV2)		
β3	1377 G>A	Val427Ile	Hybrid	0% (3FCS)	6 = intermediate	rs5921
				0% (3FCU)		
				0% (1JV2)		
β3	1980 G>A	Glu628Lys	βTD	66.5% (3FCS)	1 = variable	rs70940817
				45.6% (1JV2)		
αIIb	440 C>G	Leu116Val	β-Propeller	34.9% (3FCS)	1 = variable	
				35.8% (3FCU		
αIIb	1977 G>T	Val618Leu	Calf1	16.8% (3FCS)	1 = variable	rs7207402
αIIb	2300 C>A	Ser725Arg	Calf1	11.6% (3FCS)	9 = conserved	rs74563314
αIIb	2934 T>A	Tyr937Asn	Calf2	23.7% (3FCS)	4 = variable	rs2934

Our structural analysis revealed that the SNPs responsible for the HPAs affected nonconserved positions located on the surface of the protein, far away from the ligand binding site and not in direct contact with other subunits. In contrast, disease-causing mutations within integrins often disturb highly conserved and buried positions in the proteins' cores^38^ (Table 4, Fig. 2), which imply that they are essential for maintaining the basic structure common to all integrins. Similarly, a missense mutation leading to a rare bleeding disorder, BSS, was mapped to an evolutionary conserved and hydrophobic residue at the core of the LRR region in the GPIb-IX-V complex.^39^ In contrast, Thr145 responsible for HPA-2, located at the same repeat region (Fig. 1D), is exposed to the solvent and is evolutionary variable. Consequently, we raised the question whether immunogenicity of HPAs, as opposed to SNPs not reported to cause an immune response, is the consequence of the presence of evolutionary variable and exposed residues in HPAs. Analyses of integrin αIIbβ3 polymorphisms that were not published as immunogenic showed that some polymorphic residues are evolutionary variable and exposed similarly to HPAs, while others are buried. Only one polymorphism was shown to be evolutionary conserved, but not buried as found for missense mutations (Table 4). Statistical analysis showed that HPAs differed significantly from causing disease missense mutations in both variables: evolutionary conservation and SASA (Fig. 2). In contrast, HPAs differed from nonimmunogenic polymorphisms in SASA but not in their conservation score, suggesting that the immunogenicity is dependent mostly on the structural location of the residue. Interestingly, it was found that nonimmunogenic polymorphisms differed significantly from missense mutations in the evolutionary score but not in the SASA (Fig. 2).

Taken together, we can conclude that although disease-causing mutations and SNPs responsible for HPAs can be localized to the same domain, they differ in their evolutionary conservation and solvent exposure. Interestingly, although most HPAs result in substituting chemically dissimilar residues, they have no dramatic effects on the apparent function of the proteins. The observations that these positions are evolutionary variable and surface exposed explain their tolerance to substitutions for residues of various properties. Actually, in most cases, the amino acid of the polymorphic residue was found in equivalent positions within homologous proteins (Table 2). It is interesting to note that many of the HPA-related positions are occupied by positively charged residues. Substitutions would therefore lead to alteration of the surface electrostatic potential. This might be related to the formation of unique interfaces that lead to antigenicity and to the production of antibodies. The antigenicity of the polymorphisms seems to be dependent on the amino acid residue, as shown for HPA-1. The immunization against HPA-1a (Leu33) is known to be the most common cause of NAIT (80%) while the HPA-1b allele (Pro33) is responsible for only approximately 4% of NAIT cases. This discrepancy in the frequencies is probably due to a different antigenicity of the alleles, as shown by the observation that peptides containing the Leu33 polymorphism bind to recombinant DR52a molecules, whereas the Pro33 version does not.^12^ Similarly, HPA-5b allele is immunogenic and the HPA-5a is not (10% in NAIT compared to 0%). Although the likelihood of antigen incompatibility between mother and fetus is very high in HPA-3 and HPA-15 systems, due to their high heterozygosity rate, they are less likely to cause NAIT compared to HPA-1 and HPA-5 systems. Interestingly, HPA-1 and -5 are associated with very low evolutionary conservation (1 = variable) and very high residue SASA (Table 1).

### Some HPAs are nonlinear epitopes

Leu33 involved in HPA-1 is located in a loop of the PSI domain of integrin β3, totally exposed to the solvent in both the active and the inactive conformations of αIIbβ3 (Table 1). HPA-1 was shown to be a part of a nonlinear epitope^40^ sensitive to disulfide bond reduction or cysteine substitution in the PSI domain.^41^ This suggests that the epitope is dependent on the intact tertiary structure of its domain. Moreover, substitutions such as Arg93Gln or Pro407Ala (of HPA-7), both localized to the hybrid domain, were shown to be critical for binding of anti-HPA-1a antibodies.^42^ This indicates that the HPA-1a epitope is dependent on residues in the neighboring hybrid domain. The sensitivity of the HPA-1a epitope to polymorphisms located in neighboring region can explain its heterogeneous nature as demonstrated by the third allele Val33, which reacts with some anti-HPA-1a but not with others.^12^ In addition to HPA-1, epitopes of other HPAs were also shown to be nonlinear^43^ and affect posttranslation process: For instance, Arg636Cys (constructed HPA-8) alters the N-linked glycosylation pattern^44^ and deletion of Lys611 (HPA-14b) modifies the disulfide bond pattern, both mapped to the βTD of integrin β3. Similarly, HPA-3 determinant, caused by Ile843Ser substitution in integrin αIIb, is dependent upon O-linked carbohydrate.^45^ The dependency of the immune response on the glycosylation state can explain the heterogeneous nature of this epitope. It has been shown that some anti-HPA-3a require sialic acid or O-linked oligosaccharide residues for reactivity, whereas others do not.^45^

### Association between HPA epitopes and activation states of integrins

Integrins such as αIIbβ3 were shown to be in a bent inactive conformation on the resting PLT. After physiologic activation, the integrins shift to the extended active conformation leading to exposure of multiple new epitopes named LIBS (ligand-induced binding site).^6^ The activation mechanism of the integrins involves multiconformations of both subunits;^6^ few were recapitulated in the available crystal structures.^6^–^8^ We found some differences in the SASA of HPA-related residues in the various conformations of the β3 subunit due to structural changes in the backbone as well as different rotamers (side-chain conformation; Table 1). It is possible that structural changes and rearrangements of interdomain orientation accompanying the activation process lead to alterations in the presentation of the HPAs' epitopes. Correspondingly, some HPA-related residues are mapped to the same regions harboring the LIBS epitopes that become exposed on activation. For example, few LIBS epitopes, as well as HPAs-1, -6, -8, -11, and -14 of β3 integrin, are located in the PSI, βTD, and EGF domains.^8^ Similarly, the epitopes for HPA-3 and -9 at the αIIb integrin, as well as the epitope to the LIBS antibody PMI-1,^8^,^45^ are mapped to the same region—an unstructured loop in the calf2 domain of αIIb. This loop is cleaved during biosynthesis, thus enabling breathing movements that can shift the equilibrium toward integrin extension.^8^ The antigenic determinant of HPA-3 is similar to that of the LIBS antibody PMI-1, suggesting that the activation state might also affect exposure of the HPA-3 epitope.^45^

The rearrangements of interdomain orientation during activation impinge on the presentation of the HPA epitopes. Then again, some HPA polymorphisms can have an effect on the dynamic of the conformational changes involved in the activation process. For example, Arg633 in β3, the residue substituted in HPA-11, was shown to be important for constraining αIIbβ3 in a low-affinity state. Correspondingly, the polymorphism Arg633His can enhance the separation of domains. Interfering with interdomain interactions can increase protein flexibility and initiate the extension of the integrin.^46^ Another example is HPA-1b where a Pro33 in β3 subunit has been reported to cause hypercoagulability effects^47^ due to increased aggregability,^48^ different sensitivity to agonists,^49^ or enhancement of outside-in signaling.^50^ It is possible that the polymorphism might partially mimic the activated conformation. Our analyses show that both Arg633 and Leu33 in β3 are located close to the interface with other domains, implying that the substitutions might affect interdomain rearrangements. Taken together, these data suggest that although the residues that form the HPAs are probably not involved in stabilizing the three-dimensional fold, their surface location might shape the rigidity of the protein, and influence the interdomain orientation, thus making the protein more prone to activation.

In conclusion, our analyses provide a unique perspective to the molecular basis of amino acid substitutions leading to the formation of epitopes for auto-/alloantibodies. The HPAs involve SNPs at evolutionary variable positions located on the protein surface. These polymorphisms do not cause diseases but lead to immunologic disorders. Interestingly, some HPAs might also affect the activation process of the protein. The integrins form hetrodimers, whereas each subunit comprises multiple domains. This allows a regulation mechanism that involves multiple conformations of the protein on the cell surface. Our analyses show that some HPAs involve substitutions at positions located at the interface between domains and thereby might affect interdomain rearrangement and the activation dynamics.

## ABBREVIATIONS:

βTD: β-tail domain
BSS: Bernard-Soulier syndrome
EGF: epidermal growth factor
GP(s): glycoprotein(s)
GT: Glanzmann thrombasthenia
HPA(s): human platelet antigen(s)
LIBS: ligand-induced binding site
LRR: leucine-rich repeat
MSA: multiple sequence alignment
NAIT: neonatal alloimmune thrombocytopenia
PLT: platelet
PSI: plexins, semaphorins, and integrins
SASA: solvent-accessible surface area
SNP: single-nucleotide polymorphism
